# Comparative proteomic analysis identifies proteins associated with arbuscular mycorrhizal symbiosis in *Poncirus trifoliata*

**DOI:** 10.3389/fpls.2023.1294086

**Published:** 2023-11-23

**Authors:** Huimin Yu, Chuanya Ji, Zijun Zheng, Miao Yu, Yongzhong Liu, Shunyuan Xiao, Zhiyong Pan

**Affiliations:** ^1^ National Key Laboratory for Germplasm Innovation & Utilization of Horticultural Crops, College of Horticulture and Forestry Sciences, Huazhong Agricultural University, Wuhan, China; ^2^ Institute for Bioscience and Biotechnology Research & Department of Plant Sciences and Landscape Architecture, University of Maryland College Park, Rockville, MD, United States

**Keywords:** arbuscular mycorrhizal symbiosis, citrus, proteomic, NCER, Poncirus trifoliata, Rhizophagus irregularis

## Abstract

Citrus, one of the most widely cultivated fruit crops in the world, relies on arbuscular mycorrhizal fungi (AMF) to absorb nutrients and water from soil. However, the molecular mechanism of AM symbiosis (AMS) in citrus in general have largely been understudied. Here, using a TMT labeling proteomic approach, we identified 365 differentially expressed proteins (DEPs) in roots of *Poncirus trifoliata* (a common citrus rootstock) upon *Rhizophagus irregularis* colonization as compared with uninoculated roots, of which 287 were up-regulated and 78 were down-regulated. GO analysis revealed that the DEPs were mainly involved in biological processes such as negative regulation of endopeptidase inhibitor activity, negative regulation of endopeptidase, one-carbon metabolic process and carbohydrate metabolic process. KEGG enrichment analysis indicated that the DEPs were mainly involved in regulating metabolic pathways such as fatty acid biosynthesis, phenylpropanoid biosynthesis and carbon metabolism. Furthermore, 194 of the 365 DEPs were found to be associated with AMS-responsive genes by association analysis with our previous transcriptomes data, which highlighted the important roles of these proteins in AMS. One of the 194 DEPs, neutral ceramidase (PtNCER), was further chosen for function analysis via RNAi interfering its homologous gene *MtNCER* in a mycorrhizal model plant *Medicago truncatula*, which confirmed a positive role of NCER in AM establishment. Our results provided basic data and key candidate genes for genetic improvement of efficient nutrient uptake through AM establishment in citrus and other crops.

## Introduction

Arbuscular mycorrhizal symbiosis (AMS) is a mutually beneficial association formed between the roots of most land plants and arbuscular mycorrhizal fungi (AMF) (Lanfranco et al., 2016). This symbiotic relationship plays a vital role in enhancing plant nutrient acquisition, promoting plant growth and development, and improving plant tolerance to various biotic and abiotic stresses (Pozo and Azcón-Aguilar, 2007; Parniske, 2008; Smith and Smith, 2011). The establishment of AMS involves intricate molecular interactions between the host plant and AMF, leading to the formation of specialized structures called arbuscules, and special interfaces for bidirectional nutrient transfer called peri-arbuscular membrane (PAM) within the root cortex cells of the plant (Harrison, 1999).

Citrus is one of the most important fruit crops in the world. Due to their high heterozygosity, polyembryony and long juvenility, citrus plants usually propagate through grafting to maintain the excellent characteristics of the variety and reduce juvenility, such as *Poncirus trifoliata*, which is a popular rootstock for the citrus industry in China (Zhu et al., 2019; Huang et al., 2021). However, most citrus are cultured in hilly and mountainous areas with barren soil and few root hairs (Cao et al., 2013; Zhang et al., 2013). This seriously affects the nutrient absorption of citrus roots and the formation of fruit yield and quality. It is believed that the absorption of soil nutrients by citrus roots largely depends on symbiotic AMF (Wu et al., 2016). Indeed, studies had shown that AMF can significantly improve the growth performance of citrus (Chen et al., 2014; Xiao et al., 2014). Therefore, understanding the molecular mechanisms underlying symbiotic interaction is crucial for promoting citrus nutrient absorption. However, the molecular mechanism of AM symbiosis (AMS) in citrus was poorly studied. The study of the complex molecular dialogue between citrus and AMF remains a challenging task.

The interaction between host plants and AMF is highly complex, as the establishment of AMS involves numerous molecular and cellular developmental processes (Gutjahr and Parniske, 2013). These processes are significantly influenced by host plant root proteins responding to AMF colonization (Javot et al., 2007; Bravo et al., 2016; Floss et al., 2017). Some key proteins have been identified as essential for establishing AMS, including phosphate transporter protein PT4 (Phosphate Transporter 4), lipid biosynthetic enzyme FatM (Fat required for AMS), ABC transporters proteins STR1 (Stunted Arbuscule 1) and STR2 (Stunted Arbuscule 2), GRAS domain transcription factors such as RAM1 (Required for Arbuscular Mycorrhization1), RAD1 (Required for Arbuscule Development 1), AP2 domain transcription factors like CBX1 (CTTC Motif-Binding Transcription Factor 1), WRI5a/b/c (WRINKLED5a/b/c) and MYB (v-myb avian myeloblastosis viral oncogene homolog) domain OsPHR2 (Phosphate Starvation Response 2) transcription factor (Javot et al., 2007; Gobbato et al., 2012; Gutjahr et al., 2012; Xue et al., 2015; Bravo et al., 2017; Jiang et al., 2017; Jiang et al., 2018; Xue et al., 2018; Shi et al., 2021).

Transcriptomics analysis is a powerful tool for dissecting the interactions between plants and microorganisms. However, mRNA abundance often has a weak correlation with its corresponding protein levels, resulting in the transcriptome not fully reflecting the actual physiological role of the genes (Vogel and Marcotte, 2012). Proteomes represents the ultimate regulatory processes, providing a wealth of additional information on plant-microbe interactions (Feussner and Polle, 2015; Domingo et al., 2023). Proteomic analysis enables the systematic identification and quantification of proteins expressed in plant roots in response to AMF colonization, providing insights into the functional dynamics of the plant-microbe interaction at the protein level (Jia et al., 2019). Consequently, proteomic approaches have emerged as powerful tools for studying the molecular interactions between plants and AMF. For instance, a protective effect of AMS against arsenic stress in *Pteris vittata* was observed through the proteomic approach performed by Bona et al. (Bona et al., 2011). In addition, proteomic analysis of *Elaeagnus. angustifolia* roots revealed that the abundance of 170 proteins were significantly influenced after AMF inoculation under salt stress, which were mainly involved in the amino acid metabolism, lipid metabolism, and glutathione metabolism. (Chang et al., 2022).

In this study, proteomic analysis of *P. trifoliata* (most common rootstock of cultivated citrus) was conducted for the first time using tandem mass tags (TMT) labeling coupled with LC-MS/MS approach. 365 differentially expressed proteins (DEPs) in response to colonization by *R. irregularis* was identified. Among these DEPs, 194 were associated with differentially expressed genes (DEGs) from our previous transcriptome data. Furthermore, a reverse genetic analysis was performed to validate further the symbiotic functions of neutral ceramidase (NCER), which was conserved in mycorrhizal plant species. Our work aimed to reveal the key proteins of *P. trifoliata* in response to AMF and the majority of proteins conserved in AM-host plants. Our results provided novel basic data and insights for the underlying molecular mechanisms of AMS in citrus.

## Materials and methods

### Plant materials and mycorrhizal inoculation

Seeds of trifoliate orange [TO, *Poncirus trifoliata* L. Raf.] were surface-sterilized by washing in 1 M NaOH for 15 min and in 3% sodium hypochlorite for 10 min, followed by rinsing with distilled water. The treated seeds were soaked in water for two days and the water was changed once a day. The seeds were placed in Petri dishes and covered with sterilized gauze for 1 week in the dark (28°C), and then sown in autoclaved vermiculite and placed in a greenhouse for 1 month. Seedlings were transplanted into sterile quartz sand in 6 pots of 4 plants per pot (18 × 18 cm) and grown under greenhouse conditions (25/22°C day/night). To inoculate AMF, 400 spores of *R. irregularis* DAOM197198 were placed below the seedlings for each pot. The *R. irregularis* DAOM197198 spores were obtained from Agronutrition, Toulouse, France (https://www.agronutrition.com/en). Plants without AM inoculation were used as controls. Plants were watered twice a week with 250 ml half-strength Hoagland solution containing 20 μM phosphorus as described previously (Ji et al., 2023). After 6 weeks post-inoculation, the *P. trifoliata* roots were well colonized and arbuscules were observed, and therefore plants were sampled at this time. Lateral roots from mycorrhizal and non-mycorrhizal plants were harvested for proteomic analysis with three biological replicates, respectively labeled as AM and NM, each containing 4 plants roots.

*Medicago truncatula* genotypes A17 seeds were scarified with sulfuric acid for 4 min and surface-sterilized with 10% sodium hypochlorite for 2 min. After rinsing 5 times with sterile water, seeds were vernalized for 1 d at 4°C and germinated at 25°C for 24 h in darkness. One-day-old seedlings were transferred to new 9 cm Petri dishes containing Färhaeus medium grown at 16 h light (25°C)/8 h dark (22°C). Hairy root transformation was performed according to Limpens et al. (Limpens et al., 2004). The transformed plants were individually transplanted into cones with a vermiculite: sand (1:4 V/V) mixture. To inoculate AMF, 400 spores of *R. irregularis* DAOM197198 were placed below the seedlings for each cone. Plants were watered twice a week with modified Hoagland medium containing 20 μM phosphorus (Javot et al., 2011). Plants were harvested 5 weeks after inoculation.

### Protein extraction, digestion, and TMT labeling

The roots of citrus were ground into powders in liquid nitrogen. And then, the powders were suspended with solution containing 10% (w/v) TCA, 65mM DTT. After precipitation at -20°C for 1 h, the samples were centrifuged at 10,000 rpm at 4 °C for 45 min and the supernatant was discarded. The pellet was then lysed by SDT buffer (4% (w/v) SDS, 100 mM Tris-HCl at pH 7.6, 0.1M DTT) to extract proteins (Wiśniewski et al., 2009). Then the protein concentration was determined by using the BCA Protein Assay Kit (Beyotime). Protein digestion was performed according to procedure as described previously (Bai et al., 2019). Extracted proteins (200 μg) for each sample were digested according to the filter-aided sample preparation (FASP) procedure (Wiśniewski et al., 2009). After trypsin digestion, the peptides were desalted on a Strata X C18 SPE column (Phenomenex, Torrance, CA, USA) and vacuum dried. The peptide mixture was recombined in 0.5 M TEAB and processed according to the TMT kit (Thermo Fisher) protocol. The labeled peptides from each group were mixed and classified using AKTA Purifier 100. The dried peptide mixture was reconstituted and acidified with 2 mL of buffer A (10 mM KH2PO4 in 25% ACN, pH 3) and loaded onto a Poly SULFOETHYL 4.6 × 100 mm column (5 mm, 200 Å, PolyLC Inc., MD, Columbia, USA). Peptides were eluted at a flow rate of 1 mL/min with a gradient of 0-10% for 0-25 min, 10-20% to 25-32 min, 20-45% to 32-42 min, 45-100% to 42-47 min, and at 100% from 47-52 min and 52-60 min using buffer B (10 mM KH2PO4, 500 mM KCl, 25% ACN, pH 3). During the elution process, the absorbance value of 214 nm was monitored, and the eluted fractions were collected every 1min and lyophilized and desalted by C18 Cartridge (EmporeTM SPE Cartridges C18, Sigma, Roedermark, Germany). All samples were stored at -80°C for LC-MS/MS analysis.

### LC-MS/MS analysis

Approximately 10 μL of each fraction was injected for nano-LC-MS/MS analysis using an Q-Exactive MS (Thermo Scientific) equipped with Easy nLC1000 (Thermo Scientific). The peptide mixture was loaded onto a C18 column (Thermo Scientific EASY trap column) in buffer A (0.1% formic acid) and separated with a linear gradient of buffer B (84% ACN and 0.1% formic acid) at a flow rate of 250 nL/min. The peptides were eluted with a gradient of 0–35% buffer B from 0 to 50 min, 35 to 100% buffer B from 50 min to 58 min, and 100% buffer B from 58 min to 60 min. After chromatographic separation, the samples were analyzed by mass spectrometry (MS) using Q-Exactive mass spectrometer. The peptides were analyzed in a positive ion mode and MS spectra were acquired using a data-dependent top 10 methods dynamically choosing the most abundant precursor ions from the survey scan (300-1800 m/z) for higher-energy collisional dissociation (HCD) fragmentation. Determination of the target value was based on predictive automatic gain control (AGC). Dynamic exclusion duration was 40.0 s, survey scans were set at a resolution of 70,000 at m/z 200 and resolution for HCD spectra was set to 17,500 at m/z 200. Normalized collision energy was 30 eV and the underfill ratio was defined as 0.1%.

### Bioinformatic analysis

The LC-MS/MS data were searched against the UniProt Sus scrofa database for peptide identification and quantification using Mascot 2.2 (Matrix Science) embedded in Proteome Discoverer 1.4. Peptides were identified using the following options. Peptide mass tolerance, ± 20 ppm; fragment mass tolerance, 0.1 Da; max missed cleavages, 2; fixed modifications: carbamidomethyl (C), TMT 10-plex (N-term), TMT 10-plex (K); variable modifications: oxidation (M), TMT 10-plex (Y). The score threshold for peptide identification was set ≤0.01 false discovery rate (FDR).

DEPs were screened according to the criteria with fold change up-regulation greater than 1.5-fold or down-regulation less than 0.67-fold and a *p*-value < 0.05. OmicsBean software was used to analysis Gene ontology (GO) and Kyoto Encyclopedia of Genes and Genomes (KEGG) enrichment of these DEPs.

### Phylogenetic analyses

The deduced amino acid sequences of PtNCER was used as BLASTp queries against 22 plant species databases in Phytozome (https://phytozome-next.jgi.doe.gov/) with a cut-offs of *E*-value<1e-50. All the sequences were aligned with the MAFFT software, and a phylogenetic tree was constructed by using the Neighor-joining method. The sequences were aligned by MAFFT software. All the peptide sequences are listed in **Supplementary Table 5**.

### Plasmid construction and hairy root transformation

For promoter-GUS analysis, the *MtNCER* promoter was cloned from Medicago A17 genomic DNA, and the *MtNCERpro :* GUS vectors were constructed using Golden Gate cloning. For RNAi analysis, a ~375-bp coding sequence of *MtNCER* (*Medtr3g079190*) was cloned into the pDONR221 vector using gene-specific primers, followed by recombination into the pK7GWIWG2(II) binary vector containing DsRed as a visual selection marker (Limpens et al., 2004) using Gateway^®^ LR ClonaseTM (Invitrogen). An empty construct CHEAP-pK7GWIWG2 (II) was used as a negative control in the hairy root transformation assays (Limpens et al., 2004). All the recombinant plasmids introduced into *Agrobacterium rhizogenes* strain MSU440 and the positive Agrobacterium cells were used for hairy root transformation in *M. truncatula* A17.

### WGA and GUS staining, microscopy and quantification of AMF colonization

For promoter-GUS analysis, GUS and WGA Alexa Fluor 488 co-staining was conducted as follows. Transgenic roots were harvested 5 week after inoculation with *R. irregularis* immerged in GUS staining buffer (3% sucrose, 0.5 mM EDTA, 2 mM potassium-ferrocyanide, 2 mM potassium-ferricyanide and 1 mg/mL X-Gluc in 100 mM PBS, pH7.0), and vacuum-infiltrated for 10 min followed by incubation at 37°C in darkness for 3-5h, and then washed three times in water. The roots incubated in 10% (w/v) KOH at 90°C for 8 min, washed twice in water, socked in 1M HCl for 20 min, incubated in ×1 PBS buffer for 15 min, and then incubated in ×1 PBS containing 2 mg/L WGA 488 (Invitrogen, Waltham, MA, USA) at 4°C overnight. GUS and WGA 488 imaging were performed using a Nikon fluorescence microscope.

For RNAi analysis, the *M. truncatula* transformed roots were cleaned with water, and WGA 488 staining was conducted as indicated above. Roots were cut into 1-cm fragments, and the level of colonization was calculated according to the previously reported method of Trouvelot et al. (1986) using a Nikon fluorescence microscope.

### RNA extraction and gene expression detection

Total RNA extraction and quantitative real-time PCR were performed as previously described (Yu et al., 2023). The transcript levels of genes were normalized using the widely used housekeeping gene *Pt7g003560.1* (*PtrActin*) as a reference. For *M. truncatula* samples, the widely used housekeeping gene *Medtr6g021800* (*MtEF1α*) was used as the reference. The relative expression levels were calculated by 2^-△△ct^ with three biological replicates for each sample. All the qRT-PCR data was analyzed by T-test using a Graphpad Prism 9 software. All the primer sequences for qRT-PCR analysis were listed in **Supplementary Table 8**.

## Results

### Proteomic analysis identified 365 P*. trifoliata* proteins responsive to *R. irregularis* colonization

**Figure 1:**
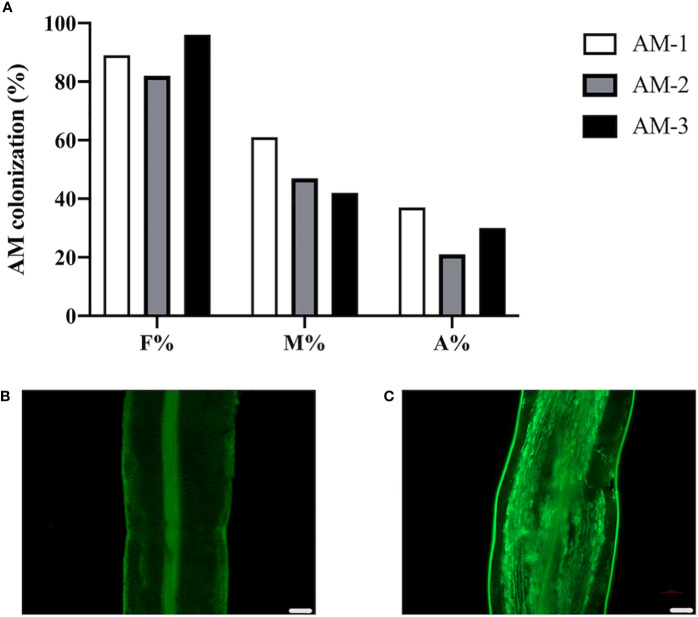
Arbuscular mycorrhizal symbiosis of *P. trifoliata*. **(A)** Quantification of mycorrhization level of three plant roots of *P. trifoliata* (named AM-1, AM-2 and AM-3 respectively) at 6 weeks post inoculation with *R. irregularis*. F%, frequency of analyzed root fragments that are mycorrhizal; M%, intensity of infection in whole roots; A%, arbuscule abundance in whole roots. **(B)** WGA staining of *P. trifoliata* roots after 6 weeks without inoculation with *R. irregularis* **(C)** WGA staining of *P. trifoliata* roots after 6 weeks inoculation with *R. irregularis* (Scale bars, 100 μm).

To investigate the change of protein levels in response to AMF colonization in the commonly used citrus rootstock *P. trifoliata*, we inoculated the roots of *P. trifoliata* with or without *R. irregularis* for 6 weeks, and then checked the AMF colonization level to ensure the AM establishment. Results showed that, in the *R. irregularis* inoculated roots, the frequency of AMF colonization (F%) in the roots was ~89%, with an arbuscular abundance (A%) of ~29%, and well-developed arbuscules were also observed (**Figures 1A, C**). As expected, no WGA488 staining (a fungal cell wall staining) was observed in the uninoculated roots of citrus rootstock (**Figure 1B**).

**Figure 2:**
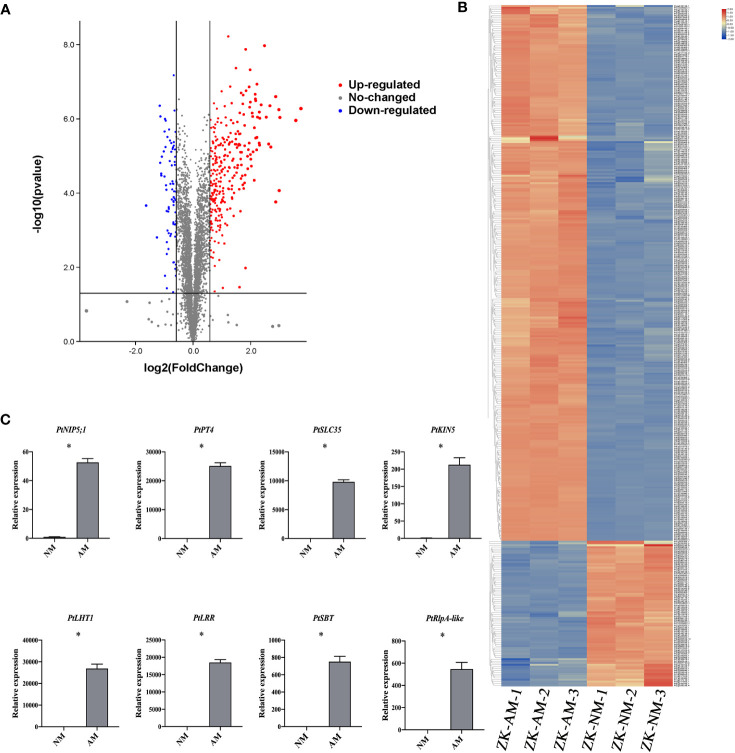
Identification of arbuscular mycorrhizal-responsive differentially expressed proteins from *P. trifoliata*. **(A)** Volcano plots of differentially expressed proteins (DEPs) between ZK-NM (roots of *P. trifoliata* uninoculation with AMF) and ZK-AM (roots of *P. trifoliata* inoculation with AMF) groups. The red dots are significantly up-regulated proteins, the blue dots are significantly down-regulated proteins, and the gray dots are no-changed proteins. **(B)** The heat-map for cluster analysis of DEPs between ZK-NM and ZK-NM. Up-regulated proteins are shown in red, down-regulated proteins are shown in blue. **(C)** qRT-PCR measurement of the expression of *PtNIP5;1 (Pt5g009680), PtPT4 (Pt9g018680), PtSLC35 (Pt2g025350), PtKIN5 (Pt1g019080), PtLHT1 (Pt2g010500), PtLRR (Pt4g009840), PtSBT (Pt8g004250)* and *PtRlpA-like (Pt5g018060)* genes in ZK-NM and ZK-AM. Expression is normalized against *Pt7g003560.1* using the 2^-△△Ct^ method. Error bars represent standard errors for three biological replicates. Asterisks represent significant differences (Student’s *t*-test: *p < 0.05).

Total proteins were extracted from the roots of citrus rootstock inoculated or uninoculated with *R. irregularis*. A proteomic approach using tandem mass tags (TMT) labeling coupled with LC-MS/MS technology was employed. A total of 5139 expressed proteins were found from 6 root samples (3 inoculated (ZK-AM) and 3 uninoculated (ZK-NM) samples, **Supplementary Table 1**). A total of 365 proteins were identified as DEPs with expression level of fold change > 1.5 and *p* < 0.05, of which 287 proteins were up-regulated and 78 were down-regulated in mycorrhizal roots relative to nonmycorrhizal controls (**Supplementary Table 2**). To obtain a visualized view of the comparative proteomic results, the up-regulated DEPs (red dots) and down-regulated DEPs (blue dots) were shown in a volcano plot (**Figure 2A**), and expression profile of each DEP was shown by a hierarchical clustering heat map (**Figure 2B**). It was worth noting that 10 proteins in roots were up-regulated over 6 folds by *R. irregularis* colonization, including Pt1g014080.1, Pt5g009680.1, Pt9g018680.1, Pt3g010560.2, Pt8g001290.1, Pt2g025350.1, Pt1g019080.1, Pt6g018480.1, Pt7g019310.1 and Pt3g011180.3. Especially, the protein Pt9g018680.1 showing the up-regulated folds (8.08 folds) was an orthologous to MtPT4 (Medtr1g028600), a symbiotic phosphate transporter strongly induced by AMF and essential for symbiotic phosphate transport (Javot et al., 2011). The protein Pt1g019080.1 showing the up-regulated folds (7.30 folds) was an orthologous to MtKIN5 (kinase 5), which was conserved for AMS identified through phylogenomics (Bravo et al., 2016).

To evaluate the reliability of the proteomic data, we examined the expression level of 8 up-regulated proteins by qRT-PCR, including PtNIP5;1 (nodulin 26-like intrinsic proteins5;1), PtPT4, PtSLC35 (solute carrier family 35), PtKIN5 (kinase 5), PtLHT1 (lysine histidine transporter 1), PtLRR (leucine rich repeat), PtSBT (subtilisin-related protease) and PtRlpA-like (rare lipoprotein A-like). The results showed that the transcription levels of all the tested proteins were also largely up-regulated in *R. irregularis* colonized samples as compared with controls (**Figure 2C**).

### Enrichment analysis of the DEPs

**Figure 3:**
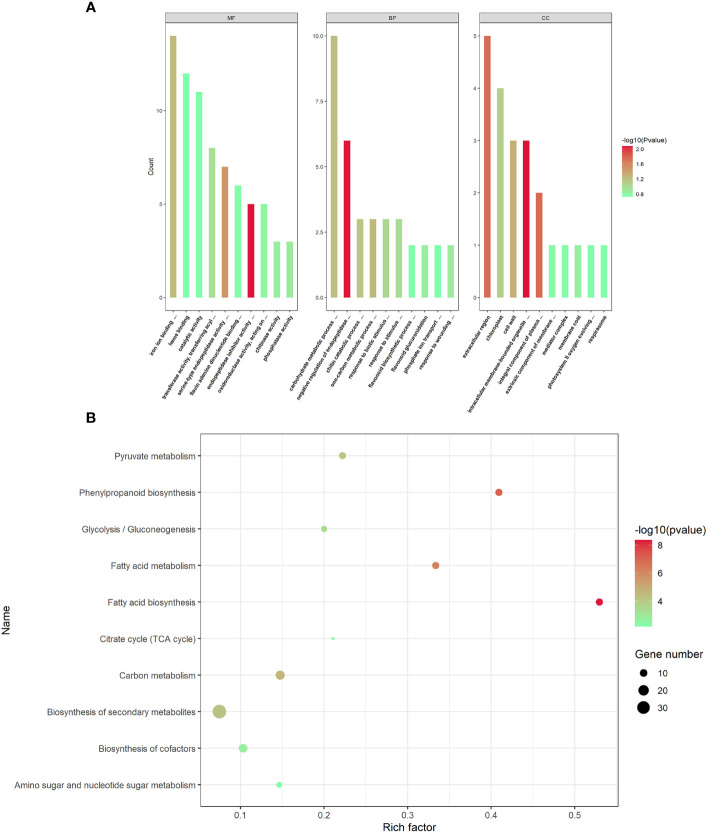
GO functional annotations and KEGG pathway enrichment analyses of the differentially expressed proteins. **(A)** The top GO terms in the biological process (BP), cell component (CC) and molecular function (MF) categories enriched with the differentially expressed proteins (DEPs). The color of the histogram represents the value of *p*-values. **(B)** The top 10 KEGG pathway enrichments of *P. trifoliata* DEPs. The size of the circle represents the number of DEPs enriched in the KEGG pathway. The color of the circle represents the value of *p*-values.

To understand the potential biological processes of the DEPs in AMS, Gene Ontology (GO) and Kyoto Encyclopedia of Genes and Genomes (KEGG) enrichment analysis were performed, respectively. GO terms were assigned to the three main categories: biological process (BP), cellular component (CC) and molecular function (MF). In BP category, DEPs were significantly enriched in negative regulation of endopeptidase activity process, one-carbon metabolic process, carbohydrate metabolic process, chitin catabolic process and response to biotic stimulus. In CC category, DEPs were notably enriched in the intracellular membrane-bounded organelle, integral component of plasma membrane, extracellular region, cell wall and chloroplast, and so on. In MF category, DEPs were observably enriched in endopeptidase inhibitor activity, serine-type endopeptidase activity, iron ion binding, transferase activity, transferring acyl groups other than amino-acyl groups, and phosphatase activity (**Figure 3A**).

The KEGG pathway enrichment analysis revealed that the 365 DEPs were significantly enriched in fatty acid biosynthesis, phenylpropanoid biosynthesis, fatty acid metabolism, carbon metabolism, biosynthesis of secondary metabolites, pyruvate metabolism, glycolysis/Gluconeogenesis, biosynthesis of cofactors, amino sugar and nucleotide sugar metabolism and citrate cycle (TCA cycle). As shown in **Figure 3B**, the most significantly enriched pathway was fatty acid biosynthesis.

### Comparison between the proteome data and our previous transcriptome data

**Figure 4:**
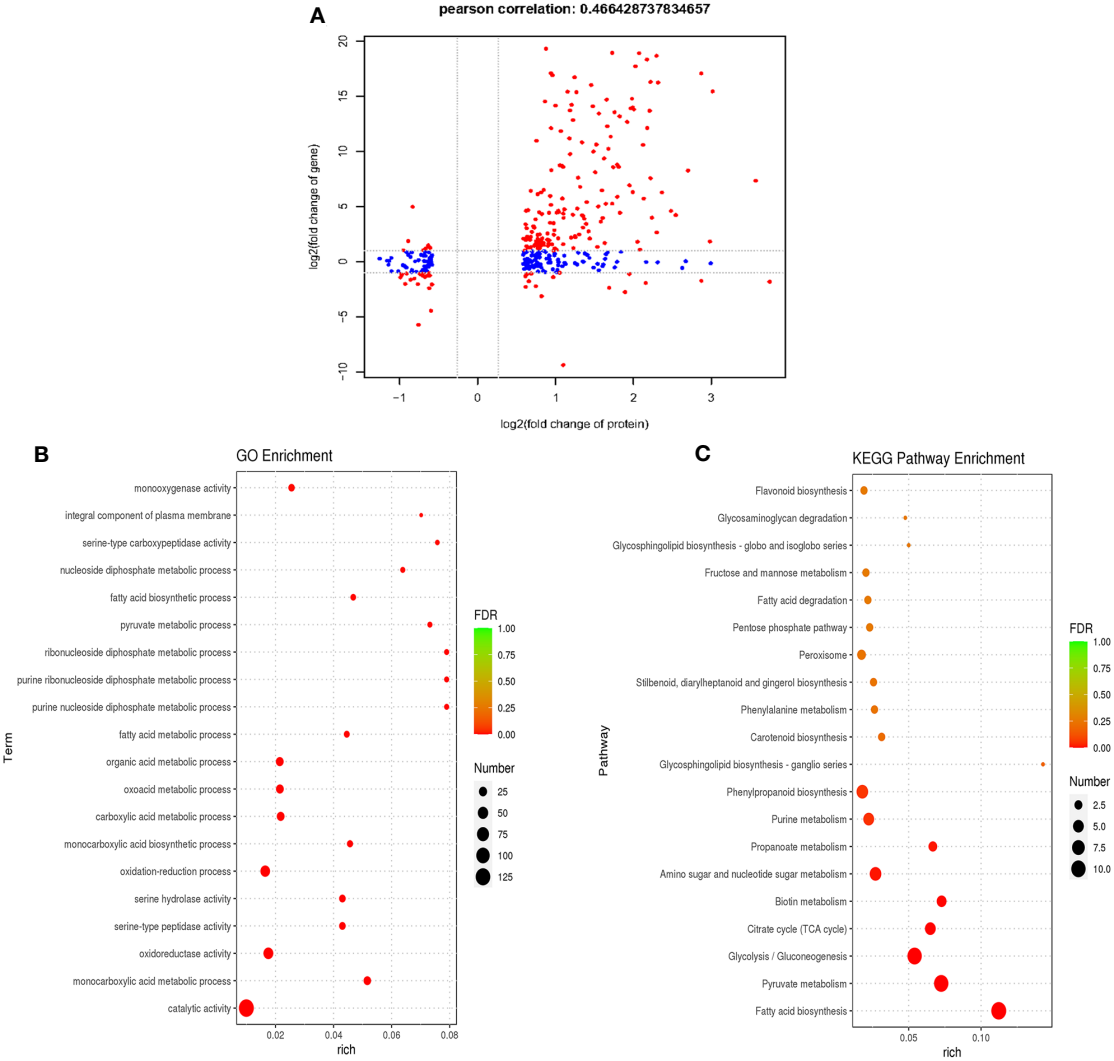
Combined analysis of differentially expressed proteins of proteome and differentially expressed genes of transcriptome. **(A)** Pearson correlation analysis of differentially expressed proteins (DEPs) and differentially expressed genes (DEGs) (Pearson correlation = 0.466). The red dots are proteins that are significantly differentially expressed in both proteome and transcriptome, the blue dots are proteins that are significantly differentially expressed in proteome. **(B)** The top 20 GO terms of *P. trifoliata* 194 key DEPs. The size of the circle represents the number of key DEPs enriched in the GO terms. **(C)** The top 20 KEGG pathway enrichments of *P. trifoliata* 194 key DEPs. The size of the circle represents the number of key DEPs enriched in the KEGG pathway. The color of the circle represents the value of False Discovery Rate (FDR).

To investigate transcriptional regulation of the formation and functioning of *P. trifoliata* during AM symbiosis, the RNA-seq of citrus rootstocks with or without *R. irregularis* were carried out in our previous study (Ji et al., 2023). A total of 3750 genes were identified as DEGs (Ji et al., 2023). To comprehensively identify the proteins involved in the formation and function of *P. trifoliata* during AM symbiosis, we carried out a correlation analysis between the proteome data and previous transcriptome data. By comparing 3750 DEGs and 365 DEPs, 194 proteins with their corresponding transcripts were successfully correlated. The 194 proteins were identified as key DEPs in response to AMF (**Supplementary Table 3**). Of these 194 key DEPs, 19 proteins associated with a set of conserved genes in five AM host plants including *Medicago truncatula*, *Poncirus trifoliata*, *Lotus japonicus*, *Oryza sativa* and *Solanum lycopersicum*, were up-regulated by AMF (**Supplementary Table 6**) (An et al., 2018). As shown in **Figure 4A**, a positive correlation between the transcript and protein abundance was observed (Pearson correlation = 0.466). Previous studies have shown that the Pearson correlation coefficient of proteome and transcriptome association analysis is about 0.40 (Vogel and Marcotte, 2012). These results further indicated that the DEPs identified by proteomic analysis of *P. trifoliata* mycorrhizal played key roles in the symbiosis between citrus and AMF.

GO enrichment analysis of the 194 key DEPs were performed to explore the potential biological function. The top 20 GO term enrichment of 194 key DEPs were shown in **Figure 4B**. In terms of the BP category, 59 of the 194 key DEPs were mainly enriched in monocarboxylic acid metabolic process, oxidation-reduction process, monocarboxylic acid biosynthetic process, carboxylic acid metabolic process, oxoacid metabolic process, organic acid metabolic process, fatty acid metabolic process, purine nucleoside diphosphate metabolic process, purine ribonucleoside diphosphate metabolic process, ribonucleoside diphosphate metabolic process, pyruvate metabolic process, fatty acid biosynthetic process and nucleoside diphosphate metabolic process. In CC category, 4 of the 194 key DEPs were primarily enriched in integral component of plasma membrane. Regarding the MF category, 132 of the 194 key DEPs were mainly enriched in catalytic activity, oxidoreductase activity, serine-type peptidase activity, serine hydrolase activity, serine-type carboxypeptidase activity and monooxygenase activity (**Figure 4B**; **Supplementary Table 4**). The KEGG pathway enrichment analysis of the 194 key DEPs was shown in **Figure 4C**. Of these proteins, 61 were significantly enriched in fatty acid synthesis, pyruvate metabolism, glycolysis/Gluconeogenesis, citrate cycle (TCA cycle), biotin metabolism, amino sugar and nucleotide sugar metabolism, propanoate metabolism, purine metabolism and phenylpropanoid biosynthesis pathways (**Figure 4C**; **Supplementary Table 5**). Among these pathways, key DEPs related to fatty acid synthesis accounted for the highest proportion at 18%.

### Functional analysis of NCER in AMS

**Figure 5:**
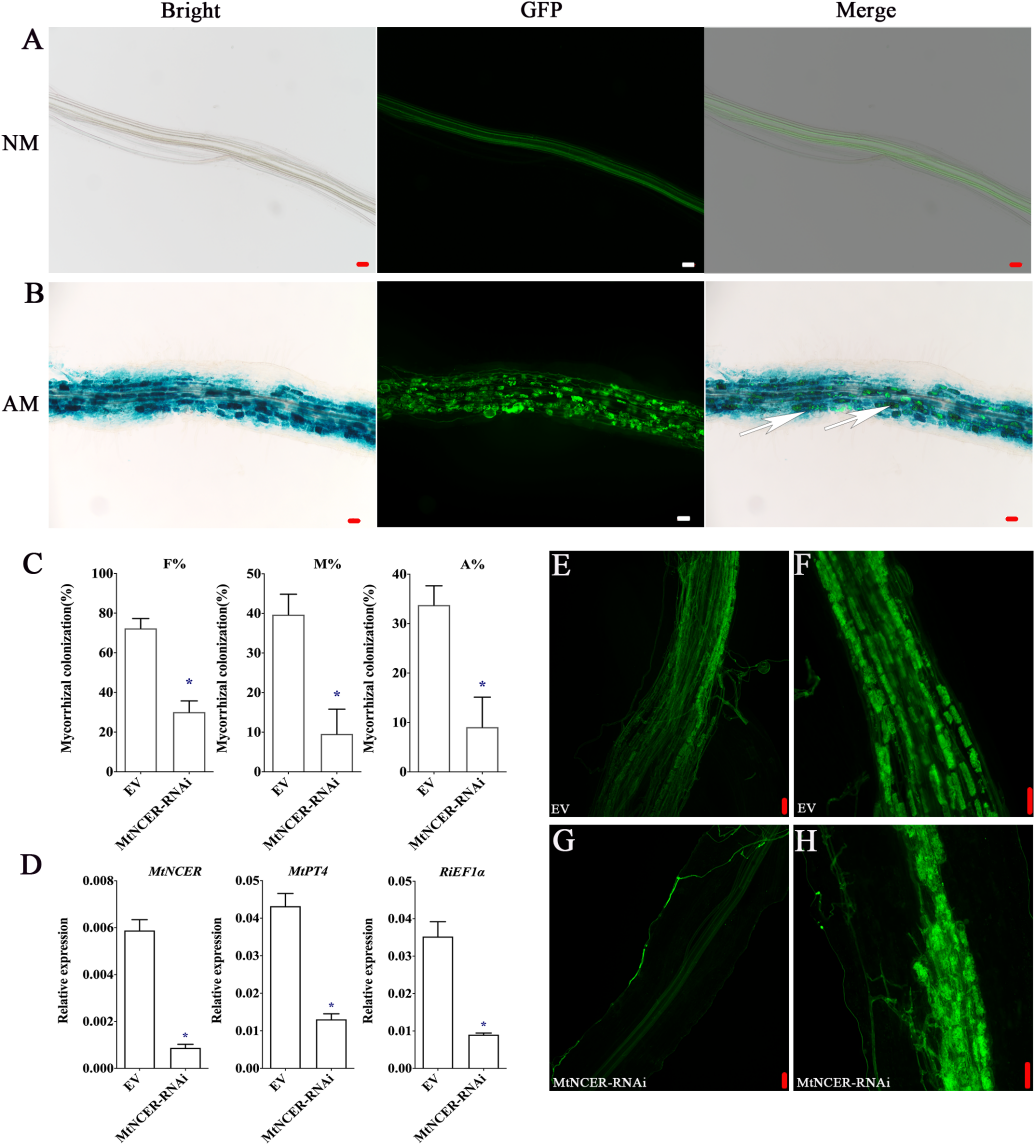
Functional analysis of NCER in arbuscular mycorrhizal symbiosis. **(A)** GUS (Bright) and WGA-Alexa Flour 488 (GFP) staining of the Medicago hairy root transformed with the *MtNCERpro : GUS* vector and uninoculated (NM) or **(B)** inoculated (AM) with *R. irregularis*. Right images showed merge of GUS and WGA-Alexa Flour 488 staining. The white arrows point to arbuscule-containing cells. **(C)** Quantification of AMF colonization levels in EV and *MtNCER*-RNAi. F%, frequency of analyzed root fragments that are mycorrhizal; M%, intensity of infection in whole roots; A%, arbuscule abundance in whole roots. **(D)** Expression levels of *MtNCER (Medtr3g079190)*, *MtPT4 (Medtr1g028600)* and *RiEF1α (XM_025321412.1)*. Expression is normalized against *MtEF1α* using the 2^-△△Ct^ method. Error bars represent standard errors for three biological replicates. Asterisks represent significant differences (Student’s *t*-test: *p < 0.05). **(E, F)** WGA-alexa488 staining of mycorrhization in roots of EV plants. **(G, H)** WGA-alexa488 staining of mycorrhization in roots of *MtNCER*-RNAi plants (Scale bar, 50 µm).

Our combined proteomic and transcriptomics of *P. trifoliata* revealed that 194 key DEPs may play key roles in the symbiosis. To further verify this result, we focused on the protein neutral ceramidase (PtNCER: Pt5g001130), which was induced by AMF and enriched in arbuscules for functional analysis. NCER is a ceramidase involved in sphingolipid metabolism. Sphingolipids are required for maintaining the arbuscular mycorrhizal endosymbionts (Moore et al., 2021). As a result of the low efficiency and long period of genetic transformation of citrus, it is difficult to test PtNCER function in citrus using RNAi or stable overexpression methods. *Medicago truncatula* was the mycorrhizal model plant. Considering that the function of genes involved in AMS is highly conserved in plants, to quickly verify the function of PtNCER, its putative homologues (MtNCER; Medtr3g079190) in *Medicago truncatula* was first verified. Previous research has found that MtNCER was highly induced in cells containing arbuscules, which were isolated by laser microdissection (**Supplementary Figure S1**) (Zeng et al., 2018). According to the ‘Noble MtGEA V3 by LIPME’ resource in the Expression Atlas (https://lipm-browsers.toulouse.inra.fr/pub/expressionAtlas/app/mtgeav3/zz.complete_dataset), the result found that *MtNCER* gene was only expressed in roots colonized with AMF (**Supplementary Figure S2**). Promoter-GUS analysis shown that GUS activity in roots expressing the *MtNCERpro :* GUS construct significantly increased upon inoculation with AMF, while no GUS activity was observed in the absence of *R. irregularis* inoculation (**Supplementary Figure S3**). Further microscopic observations showed that the GUS signals were particularly strong in root cortical cells containing arbuscules and hyphae (**Figures 5A, B**). The results indicated that the *NCER* gene was induced by mycorrhizal colonization.

Then, 70 homologous protein sequences from 20 plant species were obtained to perform phylogenetic analysis using MAFFT (**Supplementary Table 7**). Notably, phylogenetic analysis revealed that PtNCER and MtNCER proteins were clustered on clade unique to AM host plants, which further emphasizing their potentially important roles in AM symbiosis (**Supplementary Figure S4**). To investigate whether the MtNCER protein plays functional role in AMF colonization, transgenic *M. truncatula* roots expressing RNAi construct targeting *MtNCER* were generated. qRT-PCR analysis suggested that the expression of *MtNCER* were decreased by approximately 75% compared with control roots (**Figure 5D**). Reduction of *MtNCER* significantly affected colonization levels by *R. irregularis*. We observed that roots of the *MtNCER* RNAi plants exhibited a significantly lower level of both AMF colonization frequency (F%), AMF colonization intensity (M%) and arbuscule abundance (A%) compared with empty control (EV) roots (**Figures 5C, F, H**). As shown in **Figure 5E**, EV plant roots formed normal growth with an orderly arrangement of hyphae after *R. irregularis* invaded root cortex cells, while the colonization of *R. irregularis* in *MtNCER*-RNAi plant roots was inhibited, and the hyphae were observed close to the epidermal cells but did not invade (**Figure 5G**). Consistently, transcript levels of *MtPT4* (*MtPT4*, a marker gene for AMS) and *RiEF1α* (*XM_025321412.1*, a housekeeping gene representing AMF abundance in roots) were decreased in *R. irregularis*-inoculated roots of *MtNCER* RNAi compared with EV roots (**Figure 5D**). These results demonstrated that NCER was required for AMF colonization during symbiosis establishment.

## Discussion

The establishment of symbiosis between plants and arbuscular mycorrhizal fungi (AMF) is a multifaceted process involving numerous molecular and cellular developmental mechanisms within plants (Nasir et al., 2021; Ho-Plágaro and García-Garrido, 2022). These processes facilitate signal transduction and nutrient exchange, which are essential for maintaining the symbiotic relationship (Fabiańska et al., 2019; Kameoka and Gutjahr, 2022; Luo et al., 2023). Proteomics or transcriptomics are effective methods to solve biological problems. To date, transcriptomics analyses of plants involved in AMS, including *Medicago truncatula*, *Oryza sativa*, *Solanum lycopersicum* and *Poncirus trifoliata*, have identified thousands of differentially expressed genes (Gutjahr et al., 2015; Sugimura and Saito, 2017; An et al., 2018; Zeng et al., 2018). However, investigations into protein-level changes following AMF inoculation have predominantly focused on herbaceous plants such as Medicago (Talukdar et al., 2009), with limited reports available for woody plants, especially citrus. In this study, proteomic analyses of citrus rootstock *P. trifoliata* after colonization of AMF *R. irregularis* were performed, and 365 DEPs responding to AMF (287 up-regulated and 78 down-regulated) were identified for the first time. In addition, 194 key DEPs (constituting 53% of total DEPs) were found by association with DEGs previously identified through transcriptomic analysis of citrus rootstock *P. trifoliata* involved in AMS. Our combined analysis of multi-omics data provides pivotal basic data for the in-depth analysis of the molecular mechanism of AMS, and 194 overlapping DEPs provided reliable candidate genes involved in mycorrhizal symbiosis.

The absorption of nutrients and water is essential for plant growth and development. The interface for nutrient sharing between plants and AMF is provided by Periarbuscular membrane (PAM), a new plasma membrane that surrounds the arbuscules during symbiotic interaction between plants and AMF (Xue et al., 2018). The unique membrane structure of PAM is adjacent to the plasma membrane of cortical cells and represents an extension of the plasma membrane to a certain extent (Roth et al., 2019). Although it shares some proteins with the regular plasma membrane, PAM also possesses some unique membrane transporters. Membrane transporters reside in the PAM, mediating nutrient transfer from fungi to roots. The uptake of nutrients by plants depends on these membrane transporters (Pumplin and Harrison, 2009). Therefore, it is important to explore the specific membrane transporters of PAM for the molecular mechanism of AMS. Our results shown that 11 DEPs were predicted to be membrane transport, including Pt1g017100.4, Pt2g023350.3, Pt2g026610.1, Pt3g018050.1, Pt3g031530.1, Pt4g020070.1, Pt5g009680.1, Pt7g011830.1, Pt8g001290.1, Pt9g018680.1 and PtUn031030.1. Of 11 DEPs, Pt4g020070.1 and Pt9g018680.1 have high homology with MtPT4 (a phosphate transport protein), which is essential for the establishment of AMS, and is localized in PAM (Javot et al., 2007; Gutjahr and Parniske, 2013). These results indicated that Pt4g020070.1 and Pt9g018680.1 proteins were involved in phosphate transport and participated in AMF symbiotic in citrus. As one of the essential substances of AMS, water absorption plays a key role in AMS. Pt5g009680.1 (NIP5;1: nodulin 26-like intrinsic proteins5;1) has been previously identified as an aquaporin, which regulates cell membrane water permeability (Zhang et al., 2021). In our research, NIP5;1 was regulated by *R. irregularis* in citrus rootstock *P. trifoliate*, which indicted that NIP5;1 play an important role in water absorption during citrus arbuscular mycorrhizal interactions. The oligopeptide transporter (OPT) superfamily has been demonstrated to be involved in the development of root nodules in Medicago. (Seabra et al., 2012). Pt1g017100.4 belongs to OPT and is also found in DEPs, which suggested that AMS and nodule symbiosis has some similar formation mechanisms. Several studies over the past decade have reported ATP-binding cassette (ABC) transporters to be important players in the beneficial symbioses (Gutjahr et al., 2012; Kretzschmar et al., 2012; Banasiak et al., 2020). It was interesting to note that Pt7g011830.1 homologous protein AMN3 (ABC subfamily B transporter for mycorrhization and nodulation 3), common symbiotic ABC transporter, were expressed early during infection by rhizobia and AMF in Medicago (Roy et al., 2021). Among the DEPs, Pt7g011830.1, a homologous protein of AMN3, was identified as an ABC transporter. This result suggested that Pt7g011830.1 transporter is crucial to the development of AMS in citrus rootstock *P. trifoliata*. Based on the above description, a large number of transporters responding to mycorrhizal induction were identified, which also preliminarily suggested that transporters play an important role in AMS of citrus rootstock *P. trifoliata*.

AMF establish symbiotic interactions with plants, providing the host plant with minerals, in exchange for carbohydrates. Carbohydrates are required to sustain intraradical AM fungal growth (Roth and Paszkowski, 2017). Previous researches have provided evidences that plants primarily deliver carbohydrates to AMF in form of fatty acids (Jiang et al., 2017; Luginbuehl et al., 2017). During plant-AMF symbiosis, genes (such as FatM) are induced, leading to the synthesis of large amounts of fatty acids within the plant (Brands et al., 2018). Fatty acids are transported to the AMF through transport proteins (such as STR) located on the PAM (Jiang et al., 2017). In this study, the enrichment analysis of KEGG pathway of 194 key DEPs showed that fatty acid biosynthesis pathway was emerged as the most significantly altered pathway (**Figure 3B**). This result suggested that under conditions of symbiosis between AMF and woody plants, such as citrus, carbohydrates were transported to the fungus mainly in form of fatty acids. In fatty acid biosynthesis pathway, 11 proteins, including Pt4g005460.1, Pt3g007940.2, PtUn033150.1, Pt1g022510.1, Pt9g012200.1, Pt6g013760.3, PtUn010890.1, Pt8g012510.1, Pt2g001630.1, Pt5g008320.1 and Pt2g019580.2, were identified. Among these identified proteins, FatM, Pt2g019580.2 homologous protein in Medicago, was reported to be required for symbiosis (Bravo et al., 2017). The homologous protein of PtUn033150.1, which was involved in fatty acid biosynthesis, has been proved to be an essential component of the mycorrhiza-induced regulon (Martino and Crawford, 2021). PtUn010890.1 is a beta-hydroxyacyl-(acyl-carrier-protein) dehydratase (FabZ) involved in the synthesis of palmitate (C16:0), which only detected in the plasma membrane fraction of AM roots in Medicago (Aloui et al., 2018). These results suggested that proteins associated with fatty acid biosynthesis play a potential role in the maintenance of arbuscular development during AMS of *P. trifoliata*.

Sphingolipids metabolism is required for maintaining the endosymbionts of arbuscular mycorrhizal (Moore et al., 2021). Sphingolipids have long chain saturated fatty acids, creating less fluidity and a more orderly microenvironment (Gault et al., 2010). Together with cholesterol, sphingolipids establish a microstructural domain called lipid rafts. These lipid rafts rich in sphingolipids serve as the major platform recruiting proteins, achieving effective signal transduction, protein sorting, and membrane fluidity regulation, which are crucial for cellular processes (van Meer et al., 2008). Ceramide is a simple sphingolipid that usually exists in low concentrations in plants and is involved in various life activities of cells (Coant et al., 2017). In Arabidopsis, studies have shown that ceramides are cleaved by ceramidase to form a sphingosine base, which is recognized by downstream receptor kinases and activates the downstream immune response (Kato et al., 2022). The accumulation of ceramides induced the synthesis of reactive oxygen species (ROS) leading to programmed cell death (PCD) (Bi et al., 2014). The NCER protein is a unique ceramidases associated with sphingolipid metabolism (Ali et al., 2018). In our research, interfering with the NCER gene resulted in a reduced rate of AM colonization and a decrease in arbuscule abundance, without affecting arbuscule morphology (**Figures 5E-H**). We demonstrated the positive role of NCER in AM establishment. Thus, we speculated that the NCER protein affects plant immune response by influencing the accumulation of substrate ceramide during mycorrhizal interactions, thereby indirectly affecting the attachment or invasion of root epidermal cells by extraradical hyphae in the early stage of AMS. An important task in the future is to determine how NCER affects the invasion of root epidermal cells by hyphae. The mutant of NCER can be further utilized to observe whether its AMF colonization frequency and the accumulation level of ceramides are affected, thus determining the mechanism of NCER in mycorrhizal symbiosis.

In conclusion, our proteomic analysis of *P. trifoliata* roots colonized by *R. irregularis* has unveiled a set of DEPs, providing key basic data and candidate genes for uncovering the molecular mechanism of AMS in citrus. The DEPs spanned various functional categories and signaling pathways, providing valuable insights into the metabolic and cellular processes involved in AMF colonization. The positive role of NCER in the establishment of AM was confirmed, which laid a foundation for its application to the beneficial interactions between citrus plants and AMF, and ultimately to improve the effective nutrient uptake and citrus yields.

## Data availability statement

The original contributions presented in the study are included in the article/**Supplementary Material**, further inquiries can be directed to the corresponding author/s.

## Author contributions

HY: Conceptualization, Formal Analysis, Investigation, Methodology, Validation, Visualization, Writing – original draft, Writing – review & editing. CJ: Investigation, Writing – review & editing, Methodology. ZZ: Writing – review & editing, Investigation. MY: Writing – review & editing, Investigation. YL: Writing – review & editing, Supervision. SX: Supervision, Writing – review & editing. ZP: Conceptualization, Funding acquisition, Supervision, Writing – review & editing.
