# Supplementary material for: Comparative proteomic analysis identifies proteins associated with arbuscular mycorrhizal symbiosis in Poncirus trifoliata
Source: Front Plant Sci. 2023 Nov 23;14:1294086. doi: 10.3389/fpls.2023.1294086 (PMC10704097; doi:10.3389/fpls.2023.1294086)
Supplement: Supplementary file 1 [file DataSheet_1.pdf]

Supplementary Material

Supplementary Figures

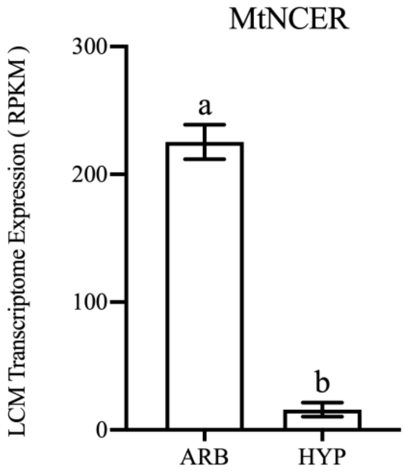

Supplementary Figure 1. Expression levels of the three *MtNCER* genes based on transcriptome analyses of laser microdissected root cells, based on Zeng et al., 2018. ARB: arbuscules. HYP: hyphae. LCM: Laser Capture Microdissection. RPKM: Reads Per Kilobase Million. Error bars represent standard errors from three biological replicates. The experiments were repeated three times with similar results. Letters indicate significant difference assessed by *t*-tests, $p<0.05$.

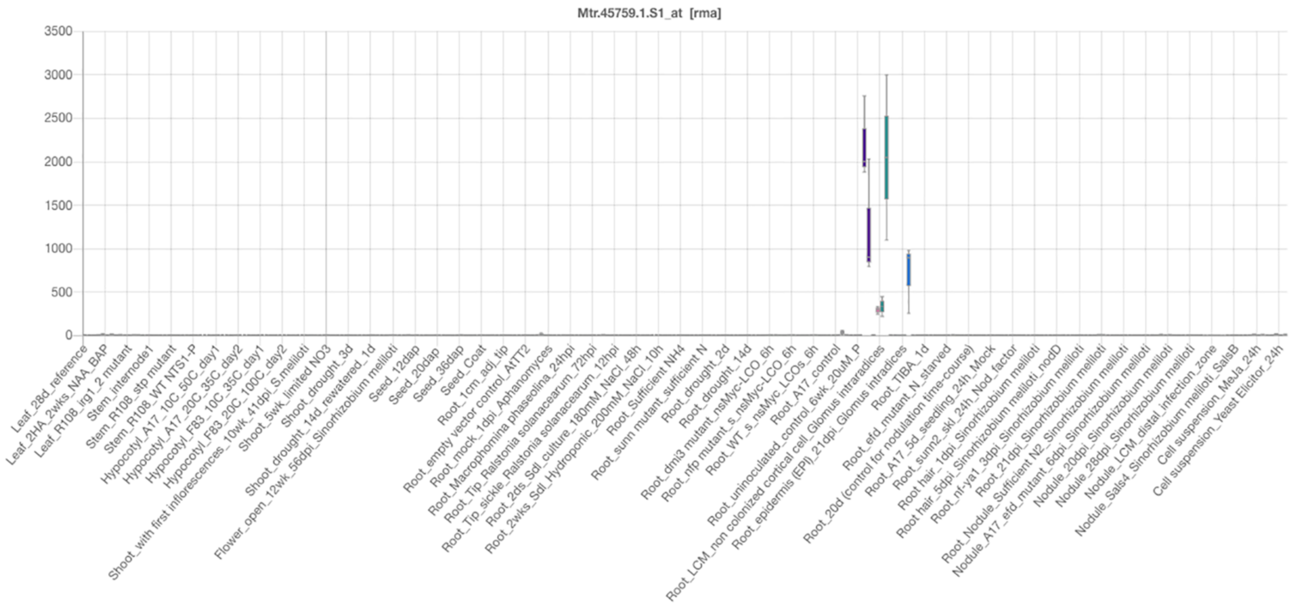

Supplementary Figure 2. Expression profiles of the *MtNCER* gene in Medicago roots upon different treatments. The expression profiles were obtained from the database ‘Noble MtGEA V3 by LIPME’.

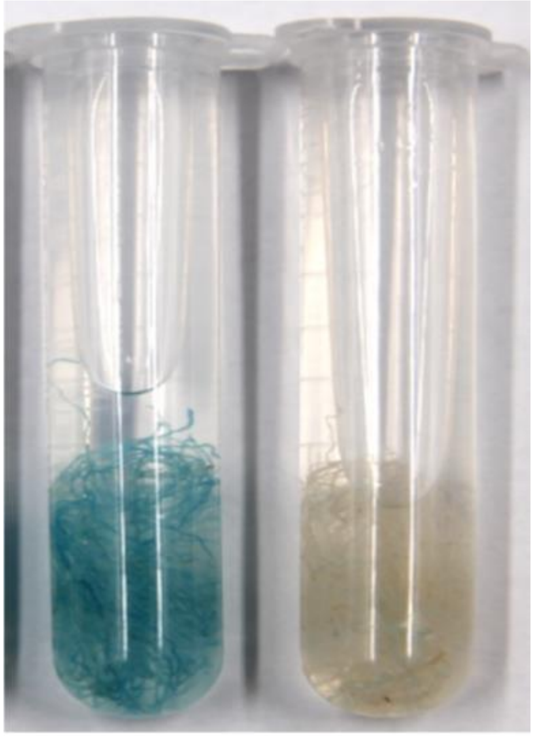

Supplementary Figure 3. GUS staining for promoter of *MtNCER*. The left tube was the Medicago hairy root transformed with the *MtNCERpro:GUS* vector and inoculated with *R. irregularis.* The right tube was the Medicago hairy root transformed with the *MtNCERpro:GUS* vector and uninoculated with *R. irregularis***.**

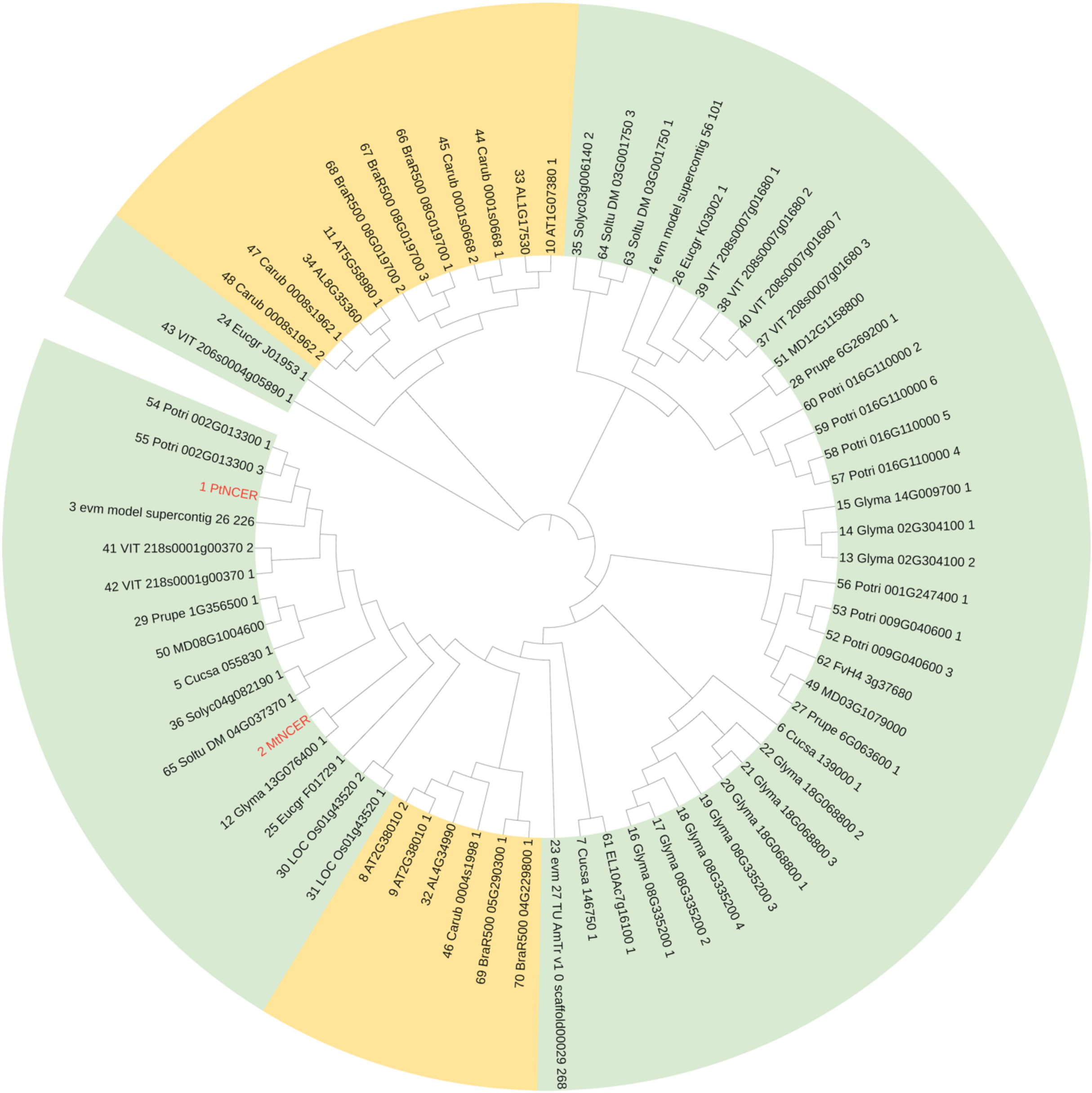

Supplementary Figure 4. Phylogenetic analysis of plant *NCER* homologous family. Yellow area labeled non-mycorrhizal plant species such as Arabidopsis and beet, green area labeled mycorrhizal plant species such as *Medicago truncatula*, *Poncirus trifoliata* and *Solanum lycopersicum*. All the peptide sequences are listed in Supplementary Table 7.
